# Elucidating the Transcriptional States of Spermatogenesis—Joint Analysis of Germline and Supporting Cell, Mice and Human, Normal and Perturbed, Bulk and Single-Cell RNA-Seq

**DOI:** 10.3390/biom14070840

**Published:** 2024-07-12

**Authors:** Ali AbuMadighem, Ofir Cohen, Mahmoud Huleihel

**Affiliations:** 1 The Shraga Segal Department of Microbiology, Immunology, and Genetics, Faculty of Health Sciences, Ben-Gurion University of the Negev, Beer Sheva 8410501, Israel; abumadig@post.bgu.ac.il; 2 The Center of Advanced Research and Education in Reproduction (CARER), Faculty of Health Sciences, Ben-Gurion University of the Negev, Beer Sheva 8410501, Israel

**Keywords:** spermatogenesis, prepubertal cancer patient boys, male fertility preservation, bulk RNA-seq, single-cell RNA-seq, male infertility: gonadotoxic treatments, chemotherapy

## Abstract

In studying the molecular underpinning of spermatogenesis, we expect to understand the fundamental biological processes better and potentially identify genes that may lead to novel diagnostic and therapeutic strategies toward precision medicine in male infertility. In this review, we emphasized our perspective that the path forward necessitates integrative studies that rely on complementary approaches and types of data. To comprehensively analyze spermatogenesis, this review proposes four axes of integration. First, spanning the analysis of spermatogenesis in the healthy state alongside pathologies. Second, the experimental analysis of model systems (in which we can deploy treatments and perturbations) alongside human data. Third, the phenotype is measured alongside its underlying molecular profiles using known markers augmented with unbiased profiles. Finally, the testicular cells are studied as ecosystems, analyzing the germ cells alongside the states observed in the supporting somatic cells. Recently, the study of spermatogenesis has been advancing using single-cell RNA sequencing, where scientists have uncovered the unique stages of germ cell development in mice, revealing new regulators of spermatogenesis and previously unknown cell subtypes in the testis. An in-depth analysis of meiotic and postmeiotic stages led to the discovery of marker genes for spermatogonia, Sertoli and Leydig cells and further elucidated all the other germline and somatic cells in the testis microenvironment in normal and pathogenic conditions. The outcome of an integrative analysis of spermatogenesis using advanced molecular profiling technologies such as scRNA-seq has already propelled our biological understanding, with additional studies expected to have clinical implications for the study of male fertility. By uncovering new genes and pathways involved in abnormal spermatogenesis, we may gain insights into subfertility or sterility.

## 1. Introduction

### 1.1. Spermatogenesis

Spermatogenesis is the process of proliferation and differentiation of spermatogonial stem cells (SSCs) to generate mature sperm. Spermiogenesis, as the subsequent phase, is the transformation of round spermatids into elongated mature spermatozoa, including the development of the tail and neck and changes in the nuclear shape [1,2,3]. This process is regulated by endocrine and paracrine factors [1,2,3,4]. Specifically, the pituitary gland secretes two primary endocrine factors: follicle-stimulating hormone (FSH) and luteinizing hormone (LH). They affect the somatic cells, the Sertoli and Leydig cells, within the testis. LH stimulates Leydig cells to secrete testosterone, which is an essential factor in the normal spermatogenesis process. FSH binds to the FSH receptors on Sertoli cells. As a result, they produce a variety of molecules, hormones, and proteins that regulate the proliferation and differentiation of the germ cells, which are present in cell-to-cell interaction and physically closed. Our knowledge of the specific biological factors regulating spermatogenesis is still partial [1,2,3,4,5].

### 1.2. Gene Expression Profiles in Spermatogenic Cells by scRNA-Seq

The biomolecular factors produced by testicular germ cells through their proliferation and differentiation stages, the cell–cell interactions between the germ cells and somatic cells, and their cellular signals in the testis that may provide the right niche for complete spermatogenesis remain unclear. Sequencing-based profiling of single-cell transcriptomes now provides a cost-effective method to survey thousands of cells to define functional subtypes and their molecular underpinning. This information naturally leads to the identification of both known and previously unknown cell types, including rare populations [6,7,8,9,10,11,12,13,14,15,16]. Single-cell RNA-sequencing (scRNA-seq) provides a comprehensive characterization of functional heterogeneity at the single-cell level and the associated marker genes. These data are essential for understanding the cell–cell interactions under normal conditions in the testis and exploring the intricate signaling patterns among germ cells and somatic/nursing cells under normal and pathological conditions [12,13,14,15,16]. scRNA-seq of mouse and human testicular tissue has revealed the normal consequence of germ cell development and identified rare cells. Key development transitions were also uncovered, and known and new candidate transcriptional regulators were associated with germ cell proliferation and differentiation in adults [14,16]. In mice, it also led to the identification of transcriptional regulators that accompany germ cell differentiation during different ages of sexual maturation [14,16]. In both mice and humans, scRNA-seq identified heterogeneous subpopulations within the spermatogonial and Sertoli cell populations. Some of these cellular subtypes were spatially mapped to histological stages [12,13,14,15,16,17]. scRNA-seq data from testicular cells of nonobstructive azoospermic (NOA) patients showed an altered transcriptional pattern in somatic cells, and the characterization of three spermatogonia subtypes, seven spermatocyte subtypes, and four spermatid subtypes. The analyses support the value of scRNA-seq for diagnosing and dissecting the mechanisms underlying male infertility [13]. The relationship between dynamic gene expression, chromatin organization (examined by scATAC-seq [18]), and accessibility in human spermatogenesis remains unknown.

**Figure 1:**
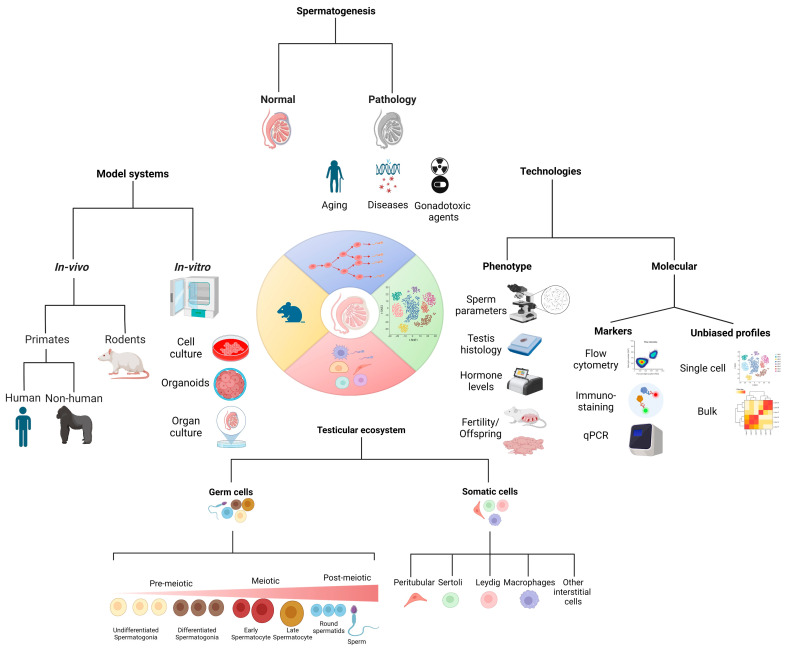
Central aspects and complementary angles for studying spermatogenesis. The study of spermatogenesis revolves around several facets in which complementary approaches are needed. This includes comparisons between normal and pathological states, such as aging, diseases, and the impact of gonadotoxic agents on male fertility (**top**); analysis of fertility phenotypes alongside their molecular profiles (**right**); consideration of not only the germ cells but also the testicular microenvironment that affects the development of spermatogenesis (**bottom**); spanning model systems used to study spermatogenesis including human samples and model systems that enable hypothesis testing with experiments (**left**).

As illustrated in Figure 1, the path to a better understanding of spermatogenesis involves traversing different research strategies that complement each other, including the model systems we profile and the technologies we use to measure phenotypic, biological, and molecular properties to shed light on both testicular germ cells and somatic cells under healthy and abnormal conditions.

## 2. Normal Physiology

### 2.1. Stages of Maturation

Spermatogenesis is the process by which sperm cells are produced following a complex regulation of SSC proliferation and differentiation. It involves several stages of SSC proliferation and maturation. The first stage includes the division and proliferation of the SSCs, followed by their differentiation into primary spermatocytes. These primary spermatocytes then undergo meiosis I to form secondary spermatocytes, which divide to form spermatids by meiosis II. The spermatids then undergo further maturation to become mature sperm cells or spermatozoa [1,2,4,6].

The main difference between these stages is that each stage involves a different type of cell division. In the spermatogonial stage, the stem cells divide mitotically to form primary spermatocytes. In the meiotic stage, the primary spermatocytes undergo meiosis to form secondary spermatocytes. Finally, in the maturation stage, the secondary spermatocytes undergo further meiosis and additional maturation stages to become mature spermatozoa [1,2,4,6].

### 2.2. Testicular Cells

Germline cells (at different stages of development – spermatogonial stem cells and all the pre-meiotic cells, meiotic and post-meiotic cells) and somatic supporting cells (Sertoli, Leydig, and peritubular cells and macrophages) form an intricate ecosystem within the testis. Germline cells provide genetic material to the next generation, while somatic supporting cells provide the optimal environment for germline cells to thrive. The cell–cell interaction between the germline cells (at different stages of differentiation) and somatic supporting cells (Sertoli cells) is mutual and leads to the development of normal spermatogenesis [7,19,20]. Without the protection and maintenance provided by the somatic cells, germline cells would not be able to survive long enough to pass on their genetic material. Similarly, without germline cells, somatic supporting cells could not produce the proteins and enzymes necessary for their survival and functionality [4,7,8,9,21].

## 3. Pathology

Numerous conditions can affect male fertility. These include genetic disorders such as Klinefelter’s Syndrome, environmental factors such as exposure to chemicals or radiation, lifestyle choices like smoking or excessive alcohol consumption, and other factors that can decrease male fertility [22]. Furthermore, hormonal disorders, structural issues, infections, medications, age, thyroid disease, celiac disease, psychological problems, and drug use can also contribute to decreased fertility in men [23,24,25].

Age: As men age, the basal membrane of seminiferous tubules tends to thicken, while the number of seminiferous epithelial cells decreases, and blood flow to the testes reduces. These changes likely affect the histological architecture, leading to a reduction in both Sertoli cells and Leydig cells [26]. These results are consistent with previous studies, which showed a decline in daily sperm production per testis with age [25]. In males, spermatogenesis continues until old age [25]. Changes in reproductive organs and hormones have also been observed with advancing age in males. There is a slight decrease in testicular size and a decrease in the inhibin B/FSH ratio, which is associated with a reduction in Sertoli cell mass [27]. FSH levels increase only slightly in men between 40 and 70 years, accompanied by a decrease in inhibin B and an increase in activin A [28].

Leydig cells become less sensitive to luteinizing hormone (LH), and LH pulse frequency decreases as a person ages. In some elderly males, this may result in rising LH concentrations, indicating the development of hypogonadism. In contrast, in others, the levels may drop due to inflammatory compounds from visceral adipose tissue or the conversion of testosterone to estradiol. Late-onset hypogonadism is relatively rare, but secondary hypogonadism associated with metabolic dyshomeostasis is more common [29,30,31].

Methylation is a common epigenetic process that has been linked to gene silencing, which may also include the acetylation of DNA or histones to adjust gene transcription and expression. A mouse model published recently demonstrated an age-related reduction in male fertility in natural and assisted reproduction cycles, along with an observed decline in blastocyst growth and implantation. In addition, the expression of four genes—Ace-1, Prm1, Prm2, and Smcp—that have been associated with decreased spermatogenesis and male infertility was seen to be altered, with a noticeable decrease in expression related to aging in males [32]. Similarly, in human donors, sperm 5-methylcytosine (5-mC) and 5-hydroxymethylcytosine (5-hmC) levels have been connected to age, which signifies decreased expression on the whole [33].

Disease: Male infertility is an often misunderstood problem, with many factors that can contribute to its occurrence. In particular, pre-testicular, testicular, and post-testicular causes can all be considered possible culprits. Pre-testicular causes can include hypothalamic or pituitary diseases, while testicular causes can range from genetic issues to environmental toxins. Post-testicular issues can include obstructive pathologies of the seminal ducts or other issues that affect the transport of sperm.

Genetic abnormalities, infections, and environmental stressors can all be causes of male infertility. Genetic abnormalities such as Klinefelter’s Syndrome [34] or mutations in the CFTR [35], DAZ [36], protamine-1 [37], GCNA [38], or SCAPER [39] genes can lead to impaired fertility or even sterility. Infections such as mumps [40] or gonorrhea [41] can lead to damage to the reproductive organs, while some sexually transmitted infections can cause scarring of the seminal ducts [42]. Environmental stressors such as exposure to pesticides [43,44], heat [45], radiation [46], or heavy metals [47] can also cause infertility in men.

### 3.1. Gonadotoxic Treatments (Chemo, Radiation, and Others)

#### 3.1.1. Effect on Adults

With the advances in cancer treatment, more and more pediatric cancer survivors are at risk of developing physical and psychological problems later in life. In particular, males may experience problems with their thyroid and sexual functions, as well as with their growth.

Recent studies have suggested that spermatogenesis can recover within 12 weeks of cessation of chemotherapy agents if they do not kill stem spermatogonia [48,49]. On the other hand, radiation or chemotherapy drugs (e.g., alkylating agents) that do kill stem cells can result in azoospermia that lasts much longer than 12 weeks [50]. Alkylating agents such as cyclophosphamide, mechlorethamine, ifosfamide, procarbazine, busulfan, and melphalan can impair spermatogenesis, as can DNA-cross-linking agents such as cisplatin [51,52,53,54].

It has been determined that the cumulative dose of a cytotoxic agent determines the length and severity of the effects on spermatogenesis. Prolonged azoospermia has been linked to high cumulative doses of cyclophosphamide [55]. It was shown that treatments with adriamycin, vinblastine, or cytosine arabinoside could exacerbate the effects of alkaline agents in causing prolonged azoospermia but only cause temporary reductions in sperm count when not used in tandem [50]. There is no definitive answer as to whether the prepubertal testis is more or less sensitive to the damage caused by chemotherapy. The Childhood Cancer Survivor Study (CCSS) has reported that only very young boys (<4 years of age at diagnosis) were more likely to be able to sire a child later in life as opposed to those who were 15–20 years of age at diagnosis, despite the prepubertal testis not displaying complete spermatogenesis [56].

Radiotherapy is a standard treatment option for numerous malignancies in men of reproductive age, and the effect of radiation depends on the amount of radiation and the delivery method. Studies have determined that doses of 0.1–1.2 Gy can cause harm to spermatogenesis, while doses of 4 Gy or greater are known to be irreversible [57]. It has been reported that, with improved radiation delivery and protection of the gonads, spermatogenesis can be restored in 9–18 months with doses up to 1 Gy, 30 months with doses up to 2–3 Gy, and five years with doses up to 4 Gy [58].

#### 3.1.2. Effect on Prepubertal Age

Alkylating agents, the primary chemotherapy used for the treatment of pediatric cancer patients, are associated with a high risk of infertility. The risk of these agents damaging DNA and inducing apoptosis depends on the type of chemotherapy/radiotherapy, combination of treatments, cumulative dose, length of treatment, age at treatment, and sex [59,60,61]. Even though the treatment of young boys and adolescents with high-dose cyclophosphamide (200 mg/kg) or total body irradiation did not affect the functionality of Leydig cells, it showed germ cell failure [62,63,64].

With the increasing survival of patients treated during childhood or adolescence by anti-cancer protocols that include mutagenic agents and even lead to the recovery of fertility, there is a concern related to possible mutations in the germ cells that may transfer to their offspring and may lead to genetic diseases [65]. It was suggested that alkylating agents and irradiation are mutagenic to germ cells [66]. It is recommended that chemotherapies distinctly affect germline mutagenicity [67].

Different studies suggest that human germline stem cells are elastic to the accumulation of spontaneous mutations [68]. On the other hand, men, after gonadotoxic treatments and recovered spermatogenesis, were concerned regarding the possible long-term effect on the genetic integrity of their gametes and the risks to the health of their future offspring [68].

The germline mutation rate in humans differs among individuals, families, and populations [69,70,71,72,73]. It is suggested that defects in genes involved in DNA repair can increase germline and somatic mutation rates [67,74], even though the defects in DNA repair may be different in the soma and the germline [67].

It was demonstrated that the long-term effect of mutation rate differences due to mutation accumulation affects reproduction and survival rates in the mouse system, and there may be a similar impact in humans [75,76]. It was suggested that paternal exposure to chemotherapy before conception may lead to germline hypermutation [67]. In contrast, it indicates that the germline is well protected from mutagenic effects, hypermutation is rare, excess mutations are relatively modest in degree, and most hypermutated individuals will not have a genetic disease [67]. On the other hand, it was shown in animal models that gonadotoxic treatments can lead to DNA damage of germ cells and increase the incidence of congenital malformations in the offspring [66,77,78,79]. However, most of the epidemiological reports did not show a significant increase in risk for offspring of cancer survivor men [65,80].

Various studies that examined the impact of radiotherapy/chemotherapy treatment of cancer patients (male, female, or both) on the development of genetic diseases in their offspring did not find a significant effect of the treatment on the development of genetic diseases [65,81]. Additional studies reported no significant differences in the proportion of offspring with cytogenetic syndromes, single gene defects, simple malformations [81], the prevalence of Down’s syndrome and Turner’s syndrome [82], abnormal karyotypes, sibling karyotype [83], risk of cancer, chromosomal disorders, and congenital malformations, in offspring of anti-cancer-treated patients at the childhood age with relation to germ cell mutations compared to control groups [81,83,84,85,86,87]. On the other hand, it was shown that treatment during the first 12 weeks of pregnancy was associated with an increased risk of congenital malformations in the fetus [88]. The epidemiological data on the effects of chemotherapy treatment of adult cancer patients on their offspring are limited. It was shown that chemotherapy affected the birth rates but not the mortality rates [80] and did not affect congenital anomalies [89] or the de novo genetic events (this study is limited to two families) [90]. However, in rodents, it was shown that anticancer drugs used separately or in combination increased preimplantation and post-implantation losses [91,92,93,94]. In addition, cyclophosphamide-treated male rats (fathers and grandfathers) have shown a rise in embryo losses [95] and postnatal mortality in litters [96,97]. Cisplatin treatment of male rats induced growth retardations in their offspring [97]. The genome of the spermatozoa is highly methylated (~80%), while the genome of the oocytes is relatively less methylated (~50%). Following fertilization, a global demethylation is performed in the parental genomes, decreasing the methylation to ~20% at the blastocyst stage. Thereafter, the methylation is restored gradually from the blastocyst stage to the gastrula stage [98]. It was demonstrated that chemotherapy treatment during adolescence led to changes in sperm DNA methylation, which was correlated with changes in sperm morphology and count that affect male fertility [99]. It is suggested that chemotherapy may induce epigenetic programming changes in human spermatogonial stem cells [99]. In addition, the ability of DNA methylation (or epigenetic changes) to cause alterations in somatic cell gene expression was reported [100]. Therefore, transmitting epigenetically modified sperm to offspring may alter their somatic cell gene activity. Thus, transmitting epigenetic information through the germline (e.g., sperm) can induce the epigenetic transgenerational inheritance of disease and phenotypic variation [100,101,102]. Accordingly, physicians should consider advising patients to cryopreserve gametes (or testicular biopsy) before chemotherapy treatments.

## 4. Model Systems to Study Spermatogenesis (Lessons Insights Gleaned from Model Systems)

To tackle the biological challenge of understanding spermatogenesis, researchers extended the study of human subjects with several model systems that are amenable to perturbations, treatments, and other functional assays that provided invaluable insights into the biological processes in healthy and pathological states.

To effectively investigate spermatogenesis, researchers utilize several distinct model systems. These biosystems are suitable for treatment, stimulation, and other assays that aid in comprehending the biological elements associated with healthy and pathological development. These functional assays are invaluable tools, as they provide researchers with a greater understanding of how spermatogenesis takes place.

Moreover, these studies are especially helpful when studying human subjects, as it is difficult to replicate the complex environment of the human body. As such, these biosystems provide an invaluable resource to better understand the steps and processes associated with spermatogenesis. Furthermore, this knowledge can be applied to the development of treatments and therapies for those suffering from infertility. Models can be used to study the effects of various treatments on spermatogenesis.

In addition to in vivo models, both in vitro and ex vivo systems provide the opportunity to study spermatogenesis in a more controlled environment. In vitro studies provide a controlled system where the factors influencing spermatogenesis can be more closely monitored and manipulated. This approach allows researchers to better understand the processes involved in spermatogenesis without the need for live animals. Similarly, ex vivo systems offer a way to study how environmental influences affect spermatogenesis without using live animal models.

### 4.1. Primates (Non-Human)

Primates offer an especially pertinent model system for investigating spermatogenesis, as they are widely considered the highest of the mammalian species. The results of studies conducted in primates are often compared to those found in humans, providing researchers with a more complete picture of the development of spermatogenesis.

Hermann et al. have demonstrated that autologous and allogeneic SSC transplants are successful in adult and prepubertal macaques rendered infertile with chemotherapy [103]. Autologous transplant resulted in donor genotype in ejaculated sperm from both adult and prepubertal recipients. Also, paternal donor origin was confirmed in 7/81 embryos produced from the sperm of one recipient, which is considered a significant achievement before clinical translation into humans [103].

Non-human primate models have been used to gain a better understanding of the role of follicle-stimulating hormone (FSH) and testosterone in regulating spermatogenesis. A study on juvenile rhesus monkeys illustrated the role of FSH and testosterone in regulating spermatogenesis, with increased testicular weight, volume, seminiferous tubule diameter, and Sertoli cell numbers [104]. The results of another study have further suggested that testosterone treatment can induce spermatogenesis in immature monkeys, some of which have produced motile sperm in their ejaculates [105].

### 4.2. Rodents

Rodents serve as a valuable model for spermatogenesis research due to their relative simplicity compared to primates, providing a more straightforward system for study. As such, rodents can provide researchers with a more straightforward view of the development of spermatogenesis, with less complexity than primates. Moreover, rodents are also more cost-effective and easier to maintain, making them a particularly useful biosystem in studies of spermatogenesis. Additionally, rodent model systems are often considered more ethical than other alternatives, further aiding in the investigation of spermatogenesis.

Using the rat system, Stanton et al. studied the proteomic of germ cells to better understand how androgens regulate meiosis [106]. Testosterone and estradiol-filled silastic implants were used to reduce androgen levels, leading to alterations in proteins involved in meiosis, apoptosis, cell signaling, oxidative stress, DNA repair, and RNA processing. Oxidized DNA adducts were observed, suggesting oxidative stress-induced DNA damage. PCNA and Ubc13 were increased, implying a role in PCNA-mediated DNA repair [106].

Another study found that testosterone withdrawal induces death by apoptosis in the testis, particularly in haploid germ cells, suggesting a role for testosterone in regulating programmed cell death alongside cell proliferation and differentiation during spermatogenesis [107].

### 4.3. In Vitro and Ex Vivo

Even though it remains challenging to generate sperm through in vitro differentiation of spermatogonial cells or ex vivo cultures of testicular fragments, any progress made towards implementing in vitro or ex vivo spermatogenesis could have a significant impact. This could be used for fertility restoration or maintenance, as the haploid spermatids produced from these processes could be used for assisted reproductive technologies, such as ICSI [108,109,110,111].

Spermatogonial stem cells (SSCs) have great potential when used as the basis for in vitro spermatogenesis [5,112,113,114,115,116,117]. Although many attempts have been made to recreate spermatogenesis in vitro using different stem cells, such as pluripotent stem cells (PSCs) [118,119] and embryonic stem cells (ESCs), the latter may not be suitable for clinical use due to the use of blastocyst embryos, while the safety of induced pluripotent stem cells (iPSCs), generated through genetic reprogramming, still needs to be investigated. Nonetheless, the most challenging process to replicate in vitro is meiosis, which is the process responsible for producing haploid spermatids through two successive meiotic cell divisions. A few studies have reported the generation of round spermatid and even sperm-like cells in vitro using human SSCs [113,117,120,121,122,123].

Constructing a testis organ in vitro can be challenging, requiring complex hormone/chemical regulation, cell-to-cell interaction, cell differentiation, and reorganizing different types of testis cells into a specific structure [124]. But simply recreating the compartmentalized testis architecture is not enough—we need to be able to replicate meiosis with proper DNA content and chromosomal recombination in order to generate viable spermatozoa from SSCs [125]. Recent reports have made considerable progress in constructing rodent and human testicular organoids [126,127,128,129,130,131,132]. Still, no one has yet been able to establish a testis organoid with accurate biomimetic structures and a complete spermatogenic cycle.

Despite their value, model systems have certain limitations. In particular, the genetic and physiological differences between species can hinder the translation of findings from model systems to humans. To alleviate this limitation, researchers have begun to employ patient-derived samples and humanized model systems. These approaches enable researchers to study spermatogenesis in a much more human-relevant context, thus allowing for a more accurate translation of findings from model systems to humans.

In addition, to alleviate these limitations, researchers are increasingly utilizing techniques such as in vivo analysis to bridge the gaps between model systems and the biology found in humans. In this way, researchers can ensure that their findings are more closely aligned with the biology of humans.

## 5. Clinical Importance (Motivation to Improve Our Understanding)

Agarwal et al. conducted an extensive study on male infertility. The results of the study revealed that there are at least 30 million cases of male infertility around the world [133]. Despite the considerable progress that has been made in understanding and managing male infertility, the etiology of more than 50% of infertile males remains unknown [134].

Investigating the roles of various cell types in the testis that regulate spermatogenesis has become an essential aspect of this research field. By elucidating the factors that influence the spermatogenesis process, we may be able to develop infertility treatments that are currently unsolved. Through scRNA-seq, researchers are now able to look at the expression profiles of different cell types within the testis [13,135,136] and identify biomarkers of fertility and infertility [137], which may open avenues for potential therapies to improve sperm production. Additionally, understanding the cellular mechanisms that regulate spermatogenesis can help to establish new strategies to protect the testes from gonadotoxic treatments such as chemotherapy/radiotherapy, which pose a risk of infertility.

To better understand spermatogenesis, researchers have begun to employ single-cell RNA sequencing-based comparative studies in humans and other species. This approach has enabled researchers to gain a better understanding of gene expression in the testis, as well as uncover potential novel therapeutic targets for male infertility. Hammoud et al. used single-cell RNA sequencing to compare and analyze the transcriptional markers of germ and somatic cells of the testes in humans, macaques, and mice, uncovering similarities and differences in expression across spermatogenesis [16]. Such cross-organism comparative studies are essential in our efforts to “align” the transcriptional profiles in model organisms to the transcriptional profiles observed in the human testicular ecosystem. Notably, to glean relevant insights from model organisms (where experiments and perturbations are possible) to humans, it is necessary to alleviate the challenge of “mapping” observations from one system to another. This “alignment” is performed with increasingly sophisticated computational strategies [138].

## 6. Technologies to Measure Phenotype and Molecular Underpinning to Study Spermatogenesis

### 6.1. Measured Phenotypes—Fertility, Sperm Parameters, Sperm Maturation, etc.

Male fertility is typically assessed via successful pregnancy and semen quality and quantity (sperm parameters). However, some phenotypic traits may be unreliable in certain environments or circumstances. Additionally, some men may be asymptomatic carriers of infertility, so reliable testing is necessary to accurately diagnose any fertility issues.

Azoospermia is a phenotypic manifestation for which at least three different types of testis histology can be present: Sertoli-cell-only syndrome (SCOS), spermatogenic arrest at different stages of germ cell maturation (spermatogonia, spermatocyte, and spermatid), and hypo-spermatogenesis. This diagnosis is performed through histological analysis of a testicular biopsy. Similar to histology, follicle-stimulating hormone (FSH) and luteinizing hormone (LH) levels, testis volume, and degree of androgenization can vary among infertile compared to fertile men.

#### Selected Molecular Markers

Flow cytometry (FCM) is a technique that has been used to study specific cells of the different stages of spermatogenesis by combining various fluorescent dyes. This has been used to investigate postnatal development in normal individuals, diagnose cases of human male infertility, and assess sperm quality [139]. In addition, FCM can be used to distinguish cell populations based on their distinctive fluorescent labels, enabling researchers to profile germ cells in different maturation stages. This method was used to enrich the spermatogonia cell population using specific markers such as GFRA-1 [140], THY-1 [141], and CD9 [142].

The most reliable techniques for assessing chromatin integrity are sperm chromatin structure assay (SCSA) [143] and Terminal deoxynucleotidyl transferase (TdT)-mediated dUTP nick-end labeling (TUNEL) [144]. SCSA is based on acid denaturation of the chromatin followed by staining with acridine orange (AO) [143].

Immunohistochemistry staining is another technique that can be used to study the cellular development of spermatogenesis and the cellular origin of growth and differentiation factors involved in this process. This technique can detect different proteins and molecules expressed within the cells of the testis, providing a visual representation of the different types of cells present in the tissue. Immunohistochemistry staining can be used to determine the relative abundance of different cell types in the testis, as well as to study the relationship and cell–cell interactions between somatic and germ cells at different stages of their development [145]. This type of staining can also provide information about the proteins involved in spermatogenesis, helping researchers understand more about the molecular processes involved in sperm production—for example, the role of leukemia inhibitory factor (LIF) in spermatogenesis [146].

### 6.2. Unbiased Profiles with Genomic Techniques-Bulk and Single-Cell RNA-Seq

Profiling of the entire repertoire of transcripts [13,136,147,148,149], proteins [106,150,151], or metabolites [150,151,152,153] provides an invaluable tool to explore in an unbiased manner the observed cellular states in the testicular tissue. Bulk RNA sequencing studies have yielded a better understanding of the molecular mechanisms behind germ cell differentiation [154,155,156,157,158,159,160,161,162,163]. Analyzing subpopulations of germ cell (primordial germ cells, gonocytes, undifferentiated spermatogonia, differentiating spermatogonia, pachytene/diplotene cells, and haploid spermatids) and somatic cell (Leydig, Sertoli, and lymphatic endothelial cells) populations extends our understanding of the regulatory molecules and functional pathways that control germ cell specification, maintenance, differentiation, and meiosis [154,155,157,159,160,162,163,164,165,166,167]. Furthermore, these studies gain insight into the transcriptomic dynamics of somatic cells across the different stages of the seminiferous tubule cycle [154,168,169,170,171,172,173,174,175].

Single-cell RNA sequencing (scRNA-seq) has the potential to improve understanding of the development of spermatogenesis at the individual cell level. By using scRNA-seq, it is possible to rapidly acquire the accurate gene expression profiles of thousands of cells in the testis. The scRNA-seq analysis enables unambiguously linking the measured transcription and the specific cell types that are expressing those genes to accurately infer transcriptional programs (as genes co-vary in expression across single cells), and to gain new insights into the development of cells, with pseudo-time computational strategies that can shed light on trajectories of cellular development and differentiation [147]. ScRNA-seq was instrumental in dissecting the spermatogenesis ecosystem, including the germ and supportive somatic cells. By providing detailed gene expression data for all cell types, scRNA-seq can map the cell populations involved in spermatogenesis, identify novel markers associated with this process, and reveal the molecular basis of spermatogenesis [13,135,147,176]. In addition, by studying variations of gene expression patterns within and between single cells, it is possible to gain insights into the cellular heterogeneity and distill a subpopulation that plays a role in spermatogenesis, as well as how these variations are related to specific cellular functions. Also, scRNA-seq can be used to track the dynamic changes in gene expression that occur throughout spermatogenesis, which can then be used to further our understanding of the different steps and mechanisms involved in this biological process [147,177].

**Figure 2:**
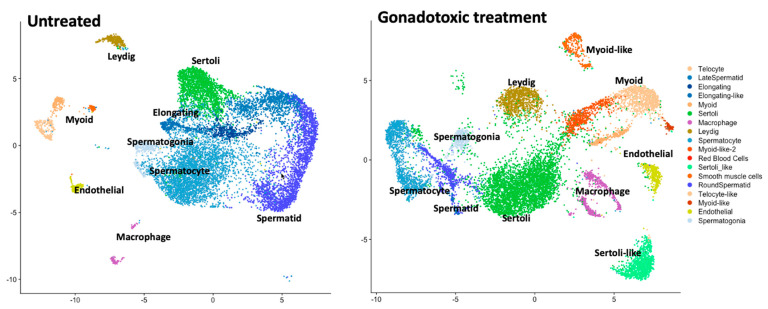
Toy example to illustrate how single-cell profiles can be used to chart the testicular ecosystem. A mock-up illustration of the transcriptional profiles of the testicular ecosystem in a murine model system, comparing the un-perturbed cell states (**left**) to those observed under gonadotoxic treatment (**right**). The expected cell types in the testicular ecosystem are denoted to convey the complexity and contribution of germline cells alongside the somatic supporting cell types.

Figure 2 is a conceptual illustration of the scRNA-seq ‘Atlas’ of the entire testicular ecosystem/niche in murine models (here, it is not based on citable data). The advancement of single-cell profiling technologies allows for comparing normal (un-perturbed) cell types and cell states and those observed under gonadotoxic treatment (perturbed). Atlases generated through scRNA-seq technologies encapsulate the transcriptional profiles from thousands of single cells embedded by dimensionality reduction into two-dimensional space. The analysis of these data forms clusters of cells, reflecting transcriptional similarity among single cells of the same cell type and state.

### 6.3. Building the Atlas of the Testicular Cellular Composition in Human and Murine Models

Single-cell RNA sequence analysis of approximately 35,000 cells revealed distinct stages of germ cell development in mice, along with new regulators of spermatogenesis, indicated by the presence of four spermatogonial cell subtypes and nine Sertoli cell subtypes. Additionally, they identified seven major somatic cell types in the testis, of which five were already known and identified, and the remaining two were previously unknown. The first unknown cluster was found to have an increased expression of markers associated with innate lymphoid type II immune cells. In contrast, the second had an elevated expression of markers, indicating a mesenchymal cell population. These findings provide valuable insight into the complex mechanisms of germ cell development in mice [12]. In addition, they reveal that re-clustering of Sertoli cells resulted in four major subtypes, which can be further divided into nine molecular clusters [12]. Also, it was observed that each major cluster contained multiple functional types, which could be present in various phases of the seminiferous epithelium cycle [12].

Lukassen et al. have presented single-cell RNA sequencing (scRNA-Seq) data of 2500 cells from the testes of 8-week-old C57Bl/6J mice [136]. This dataset includes all spermatogenic stages from preleptotene to round spermatids, as well as individual spermatogonia, Sertoli, and Leydig cells. With its comprehensive coverage of meiotic and post-meiotic stages of spermatogenesis, this dataset is ideal for various analyses such as marker discovery, network inference, and others [136]. In the study, 21 potential marker genes were identified for spermatogonia, 24 for Sertoli cells, and 20 for Leydig cells, which were significantly upregulated [136]. However, due to the limited number of spermatogonia found in this dataset and their low abundance in the testis, it is hard to tell whether this population consists of undifferentiated spermatogonia or spermatogonial stem cells (SSCs).

Chen et al. demonstrated a technique for purifying homogeneous spermatogenic cells by integrating transgenic labeling and synchronizing the seminiferous epithelium cycle, followed by single-cell RNA sequencing [178]. The transcriptomics analyses revealed distinctive markers for isolating round spermatids at particular stages, as well as differences in embryo developmental potential between early- and late-stage spermatids, indicating that the maturing of round spermatids influences embryo development [178]. This study also uncovered the presence of 9431 long noncoding RNAs (lncRNAs). The highest number and expression level of these lncRNAs occurred during diplotene and MI spermatocytes and step 6–8 spermatids [178].

Their single-cell dataset identified cell surface markers that were specific to the different clusters of round spermatids, such as CD37, CD63, CD96, and CD177 [178]. They evaluated CD63 as a candidate surface marker for distinguishing between round spermatids at different stages. CD63^high^ haploid cells were found to be enriched with step 1–2 spermatids (early spermatids), whereas CD63^low^ haploid cells were highly enriched with step 7–8 spermatids (late spermatids). Injecting CD63^low^ round spermatids (enrichment of late spermatids) with the ROSI procedure resulted in a significantly higher rate of development to blastocysts, compared to injecting CD63^high^ round spermatids (enrichment of early spermatids). Nevertheless, progression to the two-cell embryos was not statistically distinguishable between the two groups [178].

Sisakhtnezhad et al. conducted an extensive investigation of scRNA-seq-generated transcriptome data from mouse SSCs and mouse mesenchymal stem cells (MSCs). Their findings included the identification of 1584 upregulated DEGs related to reproduction, spermatogenesis, mitosis, meiosis, cell cycle regulation, and epigenetics modifications [148]. This study further introduced MAPK3, CSNK2A2, CSNK2A1, MAPK1, MAPK8, CDK1, GSK3B, CDK2, MTOR, and TEC as key upstream kinases regulating differentially expressed genes [148]. Subsequent studies have further validated the importance of these kinases for the regulation of survival, apoptosis, self-renewal, and maintenance of germline stem cells [148].

Hermann et al. analyzed the transcriptomes of 4651 spermatogenic cells from mice and identified 14 clusters of unselected spermatogenic cells. Two clusters of mice spermatogenic cells expressed known spermatogonial cell genes, such as Gfra1, Kit, Nanos3, Rhox13, Sall4, and Zbtb16 [147]. When extracted and re-analyzed, these two clusters yielded ten separate clusters of unselected spermatogonia, each distinguished by DEGs.

Clusters of unselected mouse spermatids from the mouse steady-state spermatogenic cell datasets were identified based on the expression of known spermatid-specific genes (Prm1, Prm2, Tnp1, Tnp2, and Catsper1) [147]. Following unbiased analyses, 12 distinct clusters of mouse spermatids, which were recognizable based on DEG patterns, were established. Genes associated with glycolysis, gluconeogenesis, and glucagon signaling were observed to be upregulated in mouse spermatids [147].

Sisakhtnezhad et al. used single-cell RNA sequencing to identify DEGs and their regulators in 3-day-old and 7-day-old mouse-derived single SSCs (mSSCs) [177]. Sixty-eight genes were increased, and 203 genes decreased in 7-day-old mSSCs compared to 3-day-old mSSCs, which were related to 1493 and 3077 biological processes, respectively. Moreover, DAZL, FKBP6, PAIP2, DDX4, H3F3B, TEX15, XRN2, MAEL, and SOD1 were identified as crucial elements with higher gene expression levels, which may be essential for mSSC fate and function during germ cell development [177]. For example, FkBP6 has been suggested to play a role in the self-renewal and maintenance of SSCs [177].

Kun et al. conducted a study that reveals the development of pro-spermatogonia, their descendants, and testicular somatic cells during the perinatal period in mice using single-cell RNA sequencing. This study reveals three temporally distinct pro-spermatogonial (ProSG) cell subsets, including a migratory cell population with a distinct transcriptome from the T1- and T2-ProSG stages. It also defines three undifferentiated SG subsets on postnatal day 7, two demonstrating the characteristics of newly emergent SSCs. Additionally, the development of Sertoli, Leydig, and peritubular myoid cells during the perinatal period was molecularly defined to identify candidate signaling pathways acting between germ and somatic cells in a stage-specific manner. This study provides an extensive resource for research on testicular germ and somatic cell development during the early stages of life [179].

#### 6.3.1. Spermatogonial Stem Cells (SSCs)

Male sperm production is regulated by an SSC system that requires a balance between stem cell self-renewal and differentiation. The SSCs can be categorized based on their functional properties and are part of a broader group of spermatogonia [180]. Due to modern sequencing approaches, researchers can now investigate the molecular features of different spermatogonial cell subtypes, providing potential answers to male infertility [181].

Spermatogonial cell subtypes have been the focus of research for some time. In the past, heterochromatin compaction and proliferative activity were used to classify undifferentiated human spermatogonia as A_dark_ and A_pale_ [182]. A_dark_ spermatogonia were considered reserve stem cells, while A_pale_ spermatogonia acted as progenitor stem cells [183]. However, laser-capture microdissection of A_dark_ and A_pale_ cells from testicular tissue sections revealed similar transcriptional profiles. This similarity implies that these spermatogonia may not be distinct populations but the same population in various cell cycle stages [6,184]. Single-cell RNA sequencing studies on human subjects have allowed researchers to categorize spermatogonia based on transcriptional profiles [13,16,17,135,147,149,176]. These spermatogonia can be grouped into undifferentiated (UTF1+/MKI67−) and differentiating spermatogonia (KIT+/MKI67+), with the undifferentiated group further differentiated into two subtypes, State 0 (SSC1) and State 1 (SSC2), based on gene expression signatures. Moreover, the State 0 subtype can be subdivided into three transcriptionally distinct categories: State 0 (SSC1B), State 0A (SSC1A), and State 0B (SSC1C) [17,135,149,185]. Notably, none of these transcriptional states are linked to a specific cell cycle phase [13,135,149].

Investigations of state-specific markers in testicular tissue sections and whole mounts are essential to examine the four transcriptional states of spermatogonia. Sorting and transplantation assays must be conducted to determine the stem cell potential of the transcriptional states. Although the functional effects require further investigation, several studies support the hypothesis that a population of spermatogonia expressing PIWIL4, EGR4, PLPPR3, and TSPAN33, among other markers, is the source of spermatogonial differentiation in the adult human testis [13,16,17,135,147,149,176]. This hypothesis is validated by the fact that the transcriptional profile of this cell state is similar to germ cells extricated from the testicular tissues of one-year-old boys [149,176,186].

Guo et al. [176] presented two pieces of evidence that demonstrated the plasticity of early human spermatogonia, as previously seen in various species such as Drosophila and mice [187,188,189,190]. By using RNA velocity analysis, they were able to detect the existence of a population of State 2 spermatogonia that can switch to State 1-like cells. This switch is important for maintaining homeostasis and balancing the number of early and differentiating SSC populations. Additionally, Guo et al. noted minimal modifications in open chromatin and DNA methylation along the development of spermatogonia, which may be beneficial for allowing transcriptional plasticity to occur by reducing epigenetic constraints [176].

#### 6.3.2. Sertoli Cells

Examining adult human Sertoli cells in testicular tissues has been challenging due to their size and intricate cellular structure [135,176]. As such, this cell type is underrepresented in the single-cell encapsulation process.

Despite restrictions, few studies have been able to identify groups of cells expressing established marker genes for Sertoli cells, like *AMH*, *SOX9*, and *DMRT1* [13,135,176]. These discoveries have also led to the identification of new markers like CITED1 and FATE1.

A recent study conducted by Guo et al. and Zhao et al. revealed the presence of three distinct transcriptionally types of Sertoli cells in testicular tissues from neonatal, prepubertal, and adult humans [191,192]. Referred to as stages A, B, and C, stages A and B were observed as immature Sertoli cell stages, while stage C was seen as the mature Sertoli cell stage. During human growth, the number of stage A and stage B Sertoli cells decreased until pre-puberty, while the mature Sertoli cell stage C was seen to emerge [191,192].

Zhao et al. investigated the three distinct metabolic stages of Sertoli cells during development [192]. They found that immature/stage cells had higher mRNA expression levels of mitotic and metabolism-related genes linked to oxidative phosphorylation. In comparison, mature Sertoli cells (stage C) had lower expression levels of mitotic and metabolism-related genes but higher levels associated with glycolysis. Additionally, few immature Sertoli cells were present in the adult testis [192].

While the developmental path of human Sertoli cells has been a topic of debate, a study by Guo et al. has identified two distinct immature Sertoli cell stages (immature Sertoli cells 1 and 2) that converge into the mature form at the time of puberty, without the presence of both in adult testicular tissues [191]. Further experiments will be needed to confirm this postnatal development of human Sertoli cells.

Two independent studies examining the transcriptional Sertoli cell profiles found that Sertoli cells from patients with idiopathic non-obstructive azoospermia (iNOA) showed signs of maturation arrest, with higher expression of cell cycle-associated genes and genes involved in the oxidative phosphorylation metabolism when compared with mature Sertoli cells from patients with obstructive azoospermia and normal spermatogenesis [192,193].

#### 6.3.3. Peritubular Myoid Cells

Single-cell data from Di Persio et al. identified two subtypes of adult peritubular myoid cells (PTMs), one expressing genes related to muscle contraction and the other likely involved in extracellular matrix (ECM) deposition [17]. The former expresses the muscle contraction-related genes, while the latter has high expression levels of ECM component genes and low expression levels of steroidogenic markers compared to the Leydig cell cluster [17]. These two PTMs are located in different layers surrounding the human seminiferous tubules [194].

Other single-cell studies have found a population of immature Leydig cells expressing ECM components and DLK1 in adult human testis [16,135,176]. These cells may be the same as the fibrotic PTMs that have been labeled differently [17]. Further studies are needed to evaluate and clarify the nature and function of these cells in the adult human testis.

#### 6.3.4. Leydig Cells

A recent study by Alfano et al. uncovered molecular alterations in Leydig cells of men with iNOA, showing reduced expression of mature Leydig cell marker genes and increased expression of immature Leydig cell marker genes, suggesting a delay in Leydig cell maturation [193].

A scRNA-seq study of testicular tissues from Klinefelter men revealed an increased proportion of immature Leydig cells and increased expression of genes involved in ECM deposition and regulation of the inflammatory response. Increased expression of RSPO3, a potentiator of the WNT signaling pathway, was observed, suggesting that the Leydig cells may be involved in regulating Sertoli cell maturation [195].

## 7. Limitations of the Mouse Model for Studying Human Spermatogenesis

Here are some possible limitations that should be considered when using mouse system that aim to translate to humans: (a) Species-specific differences which may affect their physiology and cellular biology; (b) Genetic variability between mouse and human—this may limit the applicability of findings; (c) Differences in testicular environment may affect the functionality and the development stages of the spermatogonial cells; (d) Differences in hormonal regulation of spermatogenesis; (e) Differences in spermatogenic wave and cell cycle; (f) Differences in spermatogenic cells and cellular markers; (g) Differences in response to gonadotoxic agents, pathologies and diseases.

## 8. Discussion, Conclusions, and Future Directions

Notwithstanding the remaining open questions and challenges in the field, in recent years, the confluence of new profiling and perturbational technologies has revolutionized our understanding of spermatogenesis.

Our review highlights the importance of adopting an integrative approach that combines various methods and types of data for advancing research in this field. We propose four key aspects in which integration is important. Firstly, investigating spermatogenesis in both healthy and pathological states, thereby gaining a broader understanding of the process where the cellular functions are intact compared with cases in which spermatogenesis is impaired. Secondly, utilizing both model systems allows for experimentation with treatments and perturbations alongside human data that reflect the biology in the clinically relevant setting. Thirdly, measuring the phenotype in several dimensions and obtaining molecular profiles using known markers and unbiased profiles allows the exploration of genes and pathways. Finally, testicular cells are examined as an ecosystem, considering both germ and supporting somatic cells to gain a more holistic understanding of the system.

We reviewed how the field had been propelled with studies using scRNAseq to chart the ‘Atlas’ of testicular tissue at the single-cell resolution level. In future studies, these and other emerging technologies are likely to facilitate the identification of new subtypes of spermatogonial cells at different stages of development and subpopulations of the testicular somatic cells under normal and pathological conditions. Importantly, these technologies may have clinical implications in the study of male fertility as we uncover new genes and pathways that are involved in abnormal spermatogenesis and lead to subfertility or sterility. Identifying those genes may lead to novel diagnostic and therapeutic strategies for precision medicine in male infertility.

Furthermore, using those technologies to identify spermatogonial and somatic cells in both physiological and pathological conditions may contribute to the optimization of the in-vitro culture systems for the generation of mature spermatozoa.
